# Transactions of the Mississippi State Medical Association

**Published:** 1883-08-20

**Authors:** 


### *TRANSACTIONS OF THE MISSISSIPPI STATE MEDICAL ASSOCIATION.*

HELD AT MERIDIAN, APRIL, 1883.

The work is neatly gotten out and contains 139 octavo pages. The membership of the Association is large, numbering 300 members. After the printed roll appears a number of able and interesting papers, as follows:

Annual Address by the retireing President, Dr. Wirt Johnson.
Annual Oration by J. W. Trimble, M. D. Subject: Intemperance as a Disease.
Recent Advances in Surgery, by Dr. S. D. V. Hill.
Malarial Hematuria, by Dr. J. E. Halbert.

Hypodermic Use of the Sulphate of Quinine, by N. L. Guice, M. D.

Vaccination, by B. A. Vaughn, M. D.

Splint for Barton's Fracture of the Radius, by John Browning, M. D. Fracture of the Femur, by the same author. External Urethrotomy, by the same author.

Rupture of the Uterus, by W. E. Todd, M. D. Typhoid Pneumonia, by the same author.

Whooping Cough, by E. L. McGehee, M. D.

Trismus Nascentium, by J. T. Hancock, M. D. Abortive Treatment of pneumonia, by the same author.

Puerperal Convulsions, by L. W. Mabry, M. D.

Chronic Hydrocephalus, by F. Kittrell, M. D. Mumps, by D. L. Phares, M. D. Surgical Case, by R. S. Toombs, M. D.

The next meeting of the Association will be at West Point, on the first Wednesday in April, 1884.

We regret that our space will not permit comment upon the above papers, some of which, however, we may glean from hereafter.

